# Upper limb position control in fibromyalgia

**DOI:** 10.1186/1471-2474-13-186

**Published:** 2012-09-24

**Authors:** Ellen Marie Bardal, Karin Roeleveld, Tonje Okkenhaug Johansen, Paul Jarle Mork

**Affiliations:** 1 Department of Human Movement Science, Norwegian University of Science and Technology, Trondheim, Norway; 2 Department of Physical Medicine and Rehabilitation, St. Olavs University Hospital, Trondheim, Norway

**Keywords:** Physiological tremor, Motor control, Shoulder joint, Elbow joint, Pain

## Abstract

**Background:**

Motor problems are reported by patients with fibromyalgia (FM). However, the mechanisms leading to alterations in motor performance are not well understood. In this study, upper limb position control during sustained isometric contractions was investigated in patients with FM and in healthy controls (HCs).

**Methods:**

Fifteen female FM patients and 13 HCs were asked to keep a constant upper limb position during sustained elbow flexion and shoulder abduction, respectively. Subjects received real-time visual feedback on limb position and both tasks were performed unloaded and while supporting loads (1, 2, and 3 kg). Accelerations of the dominant upper limb were recorded, with variance (SD of mean position) and power spectrum analysis used to characterize limb position control. Normalized power of the acceleration signal was extracted for three frequency bands: 1–3 Hz, 4–7 Hz, and 8–12 Hz.

**Results:**

Variance increased with load in both tasks (*P <* 0.001) but did not differ significantly between patients and HCs (*P >* 0.17). Power spectrum analysis showed that the FM patients had a higher proportion of normalized power in the 1–3 Hz band, and a lower proportion of normalized power in the 8–12 Hz band compared to HCs (*P <* 0.05). The results were consistent for all load conditions and for both elbow flexion and shoulder abduction.

**Conclusion:**

FM patients exhibit an altered neuromuscular strategy for upper limb position control compared to HCs. The predominance of low-frequency limb oscillations among FM patients may indicate a sensory deficit.

## Background

Fibromyalgia (FM) is a chronic pain syndrome with complex etiology, characterized by widespread muscle pain and undue muscle fatigue. Several studies indicate that FM symptoms are associated with alterations in central processing of sensory feedback [1-4].

FM patients commonly report motor problems such as poor balance and coordination, clumsiness, tremors [5], and slowness of movements [6]. However, motor problems are mainly self-reported, and few studies have investigated the characteristics of motor performance in FM patients. Studies of motor control in FM patients have shown a lower rate of change in the electromyographic mean spectral frequency of biceps brachii during sustained elbow flexion [7] and an altered activation pattern of different regions within the trapezius muscle during sustained shoulder elevation [8]. Gerdle and co-workers [9] have also found that differences between FM patients and healthy controls (HCs) in firing rate, muscle fiber conduction velocity and differential activation of the trapezius muscle are load dependent. However, neither of the abovementioned studies investigated whether the altered muscle activation patterns were accompanied by alterations in upper limb motor performance.

To optimize upper limb motor performance during fine motor tasks, the intrinsic tendency of the human motor system to oscillate (tremor) must be minimized. The amplitude of these oscillations can be used as an index of stability [10]. Limb oscillations or rhythmic muscle contractions and relaxations occur as a result of the synaptic input to the motor neuron pool [11]. Alterations in sensory information will therefore have the potential to influence the common drive and, thereby, the spectral power distribution of the limb oscillations. In addition to the neural processes causing the synchronization of motor unit firing, the amplitude and frequency content of the oscillations is dependent on mechanical properties of the limbs [12-14]. Low frequency oscillations (<7 Hz) are assumed to be influenced by voluntary, mechanical, and reflexive factors, whereas the most common range of physiological tremor (8–12 Hz) is likely to be centrally mediated [12,13,15-18]. Although the physiological correlates to the different oscillatory frequencies are not fully understood [19] the power distribution between different frequencies may still provide important information about the underlying neural processes that might induce alterations in neuromuscular control in chronic pain syndromes.

The purpose of this study was to investigate upper limb position control in patients with FM and HCs during sustained isometric contractions. Related to the motor problems commonly reported by FM patients we hypothesize that the patient group will show higher amplitude oscillations than HCs. Limb position control was tested in the elbow and shoulder joint to evaluate consistency between upper limb joints with four different load levels to reveal any dependency on mechanical load or muscle strength. Limb oscillations were recorded with accelerometers and the signal was analysed in the time domain by variance and in the frequency domain by power spectrum analysis.

## Methods

### Subjects

Fifteen female patients with FM and 13 female HCs participated in the study. Subject characteristics are presented in Table 1. The FM patients were recruited through the local FM association. Upon inclusion to the study, all patients underwent a clinical examination to verify the FM diagnosis as defined by the American College of Rheumatology [20]. The HCs were recruited from the administrative university staff. Subjects were excluded if they had: a) cardiorespiratory, cerebrovascular, neurologic, neuromuscular, endocrine, infectious, metabolic, lung, or cancer disease, b) injury that affected function, c) connective tissue disorder, d) tendinitis or capsular affection of the shoulder joint, e) high blood-pressure (i.e., systolic pressure >140 mmHg or diastolic pressure >90 mmHg) or were taking anti-hypertensive medication. Participants were also excluded if they were taking medication that may interact with neural, vascular, or muscular function or the physiological measurements to be performed (e.g., antidepressants, antiepileptics, β-blockers).

**Table 1:** Subject characteristics of fibromyalgia (FM) patients and healthy controls (HCs)

	**FM patients (n = 15)**	**HCs (n = 13)**	***P ***^**a**^
Age (years)	54.9 (8.1)	54.1 (9.3)	0.89
Height (cm)	163 (6.8)	168 (4.6)	0.056
Body mass (kg)	77.6 (12.4)	64.4 (9.7)	0.017
Upper arm girth (cm)	34.0 (4.1)	29.9 (4.2)	0.013
Epicondylar humerus width (cm)	6.7 (0.4)	6.4 (0.4)	0.059
Neck/shoulder pain before testing (VAS)	34.2 (22.8)	5.7 (10.9)	0.001
Neck/shoulder pain after testing (VAS)	41.5 (25.0)	5.8 (11.1)	<0.001
No. of tender points	14.1 (2.3)	NA	
Years since diagnosed	8.5 (6.6)	NA	
FIQ score	46.9 (19.0)	NA	

FIQ, Fibromyalgia Impact Questionnaire; NA, not applicable; VAS, visual analogue scale.

^a^ independent samples *t*-test.

Values are mean with standard deviation in parenthesis.

Intensity of neck/shoulder pain was assessed using the Visual Analogue Scale (VAS) before and after testing. Epicondylar humerus width and relaxed upper arm girth were recorded as described by MacDougall and co-workers [21]. In brief, the humerus width was measured as the distance between the lateral and medial epicondyles of the humerus, and the upper arm girth was measured at the mid-acromiale-radiale distance. Dominant arm was assessed using Edinburgh handedness inventory [22]. In addition, FM patients answered the Fibromyalgia Impact Questionnaire (FIQ) [23].

The study protocol was approved by the Regional Committee for Ethics in medical research (project no. 4.2008.2115) and all participants signed an informed consent before enrolment. The study was carried out according to the Declaration of Helsinki.

### Experimental setup and procedure

**Figure 1:**
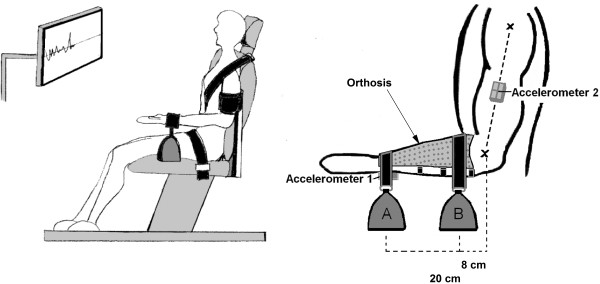
**Experimental setup.** Illustration of the experimental setup used in the elbow flexion task (**a**) and placement of accelerometers and weights (**b**). Accelerometers 1 and 2 were used to record accelerations in the elbow flexion task and the shoulder abduction task, respectively. Weight A, used in the elbow flexion task, and weight B, used in the shoulder abduction task, was attached 20 cm and 8 cm from the elbow joint rotation centre, respectively.

Subjects were placed in an adjustable and customized chair (Figure 1a). To restrict torso movements, the subjects were strapped to the chair using padded polyester strapping across the pelvic and upper body. During the elbow flexion task, the upper arm was supported by a cushion attached to the chair. Three-axis acceleration sensors (Delsys Inc., Boston, MA; range: 2 g, resolution: 0.006 g, bandwidth: 0–50 Hz) were placed at the distal part of the posterior radial surface, and on the mid-acromiale-radiale line on the lateral surface of the upper arm (Figure 1b). Sensors were placed on the dominant side to record movement of the limb segments in the perpendicular direction to the longitudinal axes of the segments.

The acceleration signal is determined by two components; the gravitational force and changes in the velocity of the accelerometer. The effect of the gravitational force is dependent on the orientation of the accelerometer in space, which forms a robust signal which we have calibrated to arm position (degrees). Since we used an isometric task, the main component of the acceleration signal was determined by arm position and was therefore used for feedback. The small variations around this main gravitational component are caused by the actual acceleration of the limb.

Force output during isometric maximal voluntary contractions (MVCs) was recorded with two force transducers (Interface Inc. Scottsdale, Arizona). The subjects wore a forearm orthosis where the force transducers were attached with non-elastic polyester bands at a standardized distance from the elbow joint rotation centre. The same sites were used for load attachments (Figure 1b). The force transducers were attached to adjustable rails at the side of the chair, ensuring that all subjects pulled vertically in the transducers.

**Figure 2:**
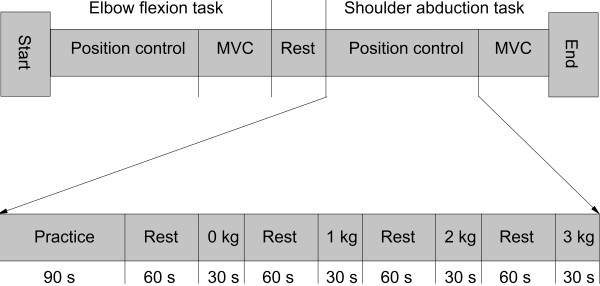
**Experimental protocol.** The protocol was identical for the elbow flexion task and the shoulder abduction task. Lower part of the figure shows the detailed time line for the position control tasks.

Acceleration of the limbs during position tasks and force output during MVCs were recorded during 90° elbow flexion and 45° shoulder abduction, respectively. The experimental protocol is presented in Figure 2. The elbow flexion task was performed unilaterally (dominant arm) with the forearm supinated, and the upper arm supported in a position of 30° shoulder abduction. The shoulder abduction tasks were performed bilaterally with the forearms pronated, and with 90° elbow flexion. Accelerometer recordings were obtained from the dominant arm only. Since the limbs were not supported in the shoulder abduction tasks, the tasks were performed bilaterally in an attempt to minimize the potential contribution from other muscles (e.g., by the oblique abdominal muscles by lateral bending). The subject’s task was to match as close as possible a constant target position of 90° elbow flexion and 45° shoulder abduction, respectively. Target position and real time feedback of arm position was provided on an 18.5" computer monitor (4:3) placed at a distance of 75 cm at eye level (Figure 1a). The monitor had a vertical range of 90 degrees and a horizontal range of 30 sec. Target position was shown as a bold horizontal line in the middle of the screen. The feedback was not enhanced to avoid overcorrection. For the shoulder abduction task the subjects received feedback for the dominant arm only. Each trial lasted 30 s, and was performed unloaded and while supporting initial loads of 1, 2, and 3 kg. The trials were separated by 1 min rest.

The subjects performed three MVCs with 3–5 s duration, separated by 1 min rest. As in the position tasks the elbow flexion task was performed unilaterally and the shoulder abduction task was performed bilaterally. The subjects were verbally encouraged to apply maximal effort during the MVCs. Maximal force level was defined by the highest achieved force level in the dominant arm across the MVCs. Isometric MVC was recorded after the position task to avoid post-activation potentiating [24].

### Data analysis

Data analysis was performed in the time domain, investigating the amount of position variability, and the frequency domain, investigating the oscillatory structure of the variability. All analysis was computed in Matlab (version 7.8.0., Mathworks, Nattick, USA).

Both the acceleration and the force signal were sampled at 1000 Hz. The first 10 s and the last 5 s of the limb stability trials were removed from the data set. The vertical component of the accelerometer signal is used in all analysis. Because of the orientation of the accelerometer on the segment, this component represents acceleration perpendicular to the longitudinal axis of the segment. The acceleration signal was filtered with an orthogonal wavelet filter. The force signal was filtered with a 10 Hz 8^th^ order Butterworth low pass filter.

In the time domain, standard deviation (SD) of the acceleration signal was calculated to indicate variance in limb position. In the frequency domain, the power spectrum was calculated using Welch’s averaged, modified periodogram method [25]. The Goertzel algorithm was used to divide the data into bins of 1 Hz, ranging from 1–25 Hz. The power within each bin was normalized to percent of total power. Normalized power were extracted from three frequency bands, i.e., a low frequency band (1–3 Hz), a middle frequency band (4–7 Hz), and a high frequency band (8–12 Hz). These frequency bands covered approximately 90% of the energy of the 1–25 Hz power spectrum. The main outcome variables from the frequency domain analysis were normalized power within the three frequency bands.

### Statistics

Descriptive statistics including mean, standard deviation (SD), and 95% confidence interval (95% CI) were calculated for the main outcome variables. A mixed design repeated measures ANOVA (2x4) was used to investigate the effect of group (two levels; HCs and FM patients) and load (four levels; 0, 1, 2, and 3 kg) on SD of mean limb position and normalized power within the three frequency bands. Group by load interactions were also tested. When the assumption of sphericity was violated, significance was adjusted using the Greenhouse-Geisser method. Independent samples tests were used to investigate between-group differences within the different load levels. The independent samples *t*-test was used to test differences between groups for normally distributed data while the Mann–Whitney *U* test was used to test group differences for non-normally distributed data (3 of 46 parameters were not normally distributed). Spearman’s ρ was used to assess correlations between anthropometric variables and normalized power within the three frequency bands. All tests were performed two-tailed and statistical significance was accepted at *P <* 0.05 for all comparisons.

## Results

The FM patients had similar age, but were heavier than the HCs, had a larger upper arm girth, and higher self-reported neck/shoulder pain (Table 1). All subjects performed the required tasks without difficulties, and the FM patients did not report any significant change in shoulder/neck pain from before to after the testing (*P =* 0.15).

### Time domain

**Figure 3:**
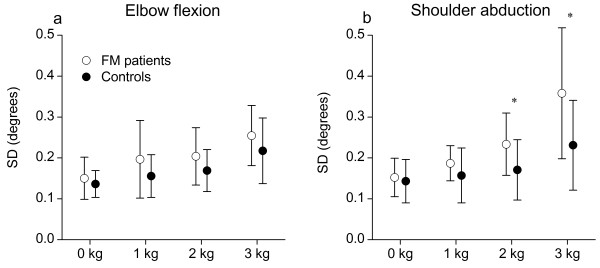
**Limb position stability.** Mean standard deviation (SD) of the acceleration signal calibrated to angle (degrees) during sustained elbow flexion (**a**) and shoulder abduction (**b**) at four different load levels (0, 1, 2, and 3 kg). Error bars indicate 95% confidence interval.

Figure 3 shows the SD of mean limb position during sustained elbow flexion (a) and shoulder abduction (b) at different load levels. There was a significant main effect of load level in elbow flexion (*P <* 0.001) and shoulder abduction (*P <* 0.001), but no main effect of group (*P >* 0.17 for both joints) or interaction between group and load level (*P >* 0.13 for both joints). Although there was no main effect of group we observed that SD of limb displacement differed significantly between groups for the shoulder abduction task supporting 2 kg (*P =* 0.04) and 3 kg (*P =* 0.03).

### Frequency domain

**Figure 4:**
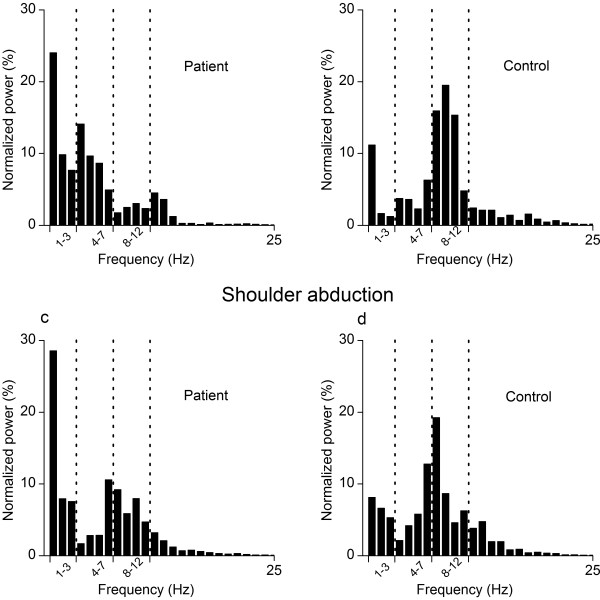
**Example of the distribution of normalized power of acceleration.** The power distribution is shown during unloaded elbow flexion (**a**, **b**) and shoulder abduction (**c**, **d**) for one fibromyalgia patient (**a**, **c**) and one healthy control (**b**, **d**). Bars indicate mean normalized power within bins of 1 Hz while dotted vertical lines denote the frequency bands 1–3 Hz, 4–7 Hz, and 8–12 Hz.

Approximately 90% of the total energy between 1–25 Hz was covered by the three frequency bands 1–3 Hz, 4–7 Hz, and 8–12 Hz. Figure 4 shows typical frequency distributions of the acceleration signal for one FM patient (a, c) and one HC (b, d) during unloaded elbow flexion and shoulder abduction. There was a tendency that the FM patients had a higher percentage of the total power covered by the three frequency bands; however, this was only significant for the elbow flexion task when supporting 1 kg (FM, 94.2%, SD 3.9 vs HCs, 86.5%, SD 11.5, *P* =0.031) and 2 kg (FM, 92.3%, SD 5.3 vs HCs, 84.3%, SD 11.7, *P* =0.036). There were no significant differences in the absolute power in the 1–25 Hz range between the groups for any of the joints or load levels (*P >* 0.28 for all comparisons).

**Figure 5:**
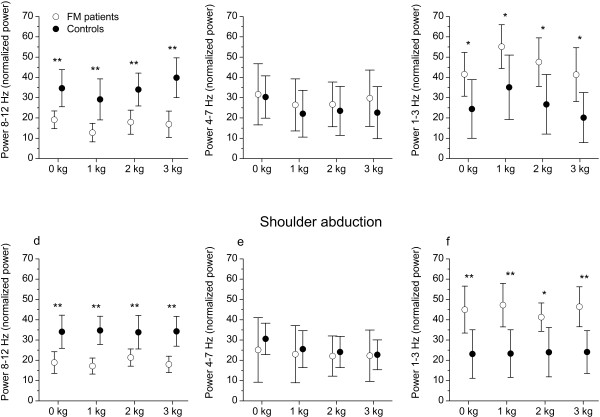
**Power in the high-, middle-, and low frequency band.** Mean normalized power in the 8–12 Hz band (**a**, **d**), 4–7 Hz band (**b**, **e**), and the 1–3 Hz band (**c**, **f**) for elbow flexion (**a**, **b**, **c**) and shoulder abduction (**d**, **e**, **f**) at four different load levels (0, 1, 2, and 3 kg). Error bars indicate 95% confidence interval. *P < 0.05; **P < 0.01.

Figure 5 shows mean normalized power in the high (a, d), middle (b, e), and low (c, f) at the four different load levels for elbow flexion (a-c) and shoulder abduction (d-f). For both elbow flexion and shoulder abduction there was a main effect of group within the low (*P <* 0.03 for both joints) and high frequency band (*P <* 0.001 for both joints), but not in the middle frequency band (*P >* 0.21 for both joints). A main effect of load level was found within the low (*P =* 0.002) and high (*P =* 0.01) frequency band during elbow flexion but not for shoulder abduction (*P >* 0.10 for both frequency bands). There was no interaction between group and load within any of the frequency bands (*P >* 0.33 for all tests).

Group wise comparisons within each load level showed that the FM patients had a significantly higher normalized power located in the low frequency band, and a significantly lower normalized power in the high frequency band compared with the HCs (Figure 5). This result was consistent for all load levels and for both elbow flexion and shoulder abduction. No significant group differences were found in the middle frequency band (*P >* 0.17 for all comparisons).

Normalized power in the three frequency bands was independent of age, neck/shoulder pain (i.e., before and after testing), years since FM diagnosis, FIQ score, number of tender points, epicondylar humerus width, and force output during MVC. Arm girth was significantly and negatively correlated with normalized power in the high frequency band and positively correlated with the low frequency band (15 of 32 correlations signinficant). The significant correlations were evenly distributed among groups, load levels, and joints. (range: FM Spearman’s ρ = 0.50 to 0.69, *P =* 0.04 to 0.004, HCs Spearman’s ρ = 0.54 to 0.64, *P =* 0.049 to 0.016).

### Maximal voluntary contraction (MVC)

The FM patients had significantly lower force output during MVC of shoulder abductors compared to HCs (FM, 13.1 kg, SD 3.6 vs HCs, 19.1 kg, SD 5.9, *P =* 0.003), but not during MVC of elbow flexors (FM, 17.3 kg, SD 3.7 vs HCs, 19.2 kg, SD 3.0, *P =* 0.16). As a consequence, the FM patients used a higher percentage of maximal voluntary force during the loaded shoulder abduction task compared to the HCs (*P =* 0.001 for all load conditions).

## Discussion

The main finding of this study was a large and consistent difference in the distribution of normalized power of upper limb oscillations between FM patients and HCs in sustained position tasks of the elbow and shoulder joint. Upper limb oscillations in the FM patients showed a strong dependency towards lower frequencies during both elbow flexion and shoulder abduction. Moreover, this difference was consistent across different loading conditions. Even though there were no overall significant differences between FM patients and HCs in variance of limb stability (time domain), neither during sustained elbow flexion nor shoulder abduction, the FM patients had a significantly higher variance in the shoulder abduction task when supporting 2 and 3 kg than HCs. Based on these results we can neither confirm nor reject the hypothesis that FM patients have higher amplitude oscillations compared with HCs.

### Time domain

The non-significant difference in steadiness between FM patients and HCs is in line with results of limb stability found in other chronic pain conditions, such as subacromial impingement syndrome [26], and knee osteoarthritis [27]. In contrast, a significant decrease in head steadiness during a 10 s isometric cervical flexion task has been found in patients with chronic neck pain [28]. However, the adjustment period at the start of this task was not removed prior to analysis and initial gross adjustments may have influenced the result. It should be noted that the abovementioned studies investigated force control while the current study investigated position control. Force control differs from position control in several important parameters such as muscle coactivation [29], reflex responsiveness to afferent feedback [30], recruitment pattern of the motor unit pool [31], and joint stiffness [32]. Hence, the results of force control studies cannot directly be compared with our results, but are rather used as an indication of system stability in chronic pain conditions. It should also be noted that various chronic pain conditions may differ in terms of the possible effects on motor control characteristics, making generalization of findings across conditions difficult. Nevertheless, both our and the previous studies referred to above indicate that isometric limb stability is only mildly influenced by FM or other chronic musculoskeletal pain conditions.

### Frequency domain

Compared to HCs, FM patients showed a preponderance of normalized power in the low frequency band. Tremor is most often measured with an accelerometer, but is initially a displacement. Even though the power of an acceleration signal is elevated in high frequencies, the displacement amplitude represents a small share of the total displacement signal [33,34]. A recent study of index finger oscillations showed that removal of frequencies corresponding to our low frequency band (i.e., 1–3 Hz) resulted in a 56% reduction of the original amplitude [34], which emphasize the importance of including low frequency oscillations when studying limb position control. Although the origin of the frequencies between 1–3 Hz are not completely understood they seem to be influenced by voluntary control [16], the processing and integration of visual feedback [16,35,36], and stress hormone level [37].

The task in the present study was to match a given limb position using real-time visual feedback. The use of visual feedback during limb stability tasks seems to increase the amplitude of low frequency oscillations [15,38,39]. It has also been demonstrated that motor disorders, low level of fine motor skills, age, and inability to voluntarily modulate tremor cause an additional increase in the amplitude of low frequency limb oscillations when using visual feedback to reduce limb oscillations [15,16,39,40]. Our finding of increased normalized power in the low frequency band among FM patients may therefore relate to the motor problems commonly reported by these patients [5,6,41].

It has been suggested that subjects with low motor skills are more dependent on sensory feedback such as vision and proprioception to update their movement plan/strategy [39]. Vaillancourt et al. (2001) on the other hand propose that increased low frequency oscillations during force control in Parkinson patients are associated with hyperactive feedback loops [15]. Increased low frequency oscillations are often accompanied by an increase in overall time-domain variance [37]. In the current study there was a mild and non-significant difference in motor performance between FM patients and HCs indicated by time-domain variance. Our study, however, indicates that maintenance of a stable limb position is achieved by different control strategies in FM patients and HCs. The enhanced power in the low frequency band among FM patients may indicate a larger dependency upon visual feedback to reduce low frequency oscillations. To examine the effect of visual feedback in the FM group, further studies should examine the frequency distribution when visual feedback is not available.

Alternatively, the enhanced low frequency power among FM patients may relate to a deficit in the sensory feedback system. Several indications of sensory deficits are observed in FM, including amplification of sensory input [1,2,4], disruption of somatosensory processing, mismatch between sensory feedback and motor output [42], and impaired visual and vestibular control [43]. These sensory alterations are suggested to be related to acceleration of age-related changes in the grey substance of the brain [44], especially in pain related areas [45], but also in motor areas [46].

Alteration in sensory information might lead to higher reliance on muscle coactivation to maintain joint stiffness [32]. While coactivation leads to high metabolic energy consumption, the use of sensory feedback is more energy efficient [47,48]. The coactivation strategy seems to reduce the ability to perform high-acceleration movements and is often observed in elderly [40]. Although we observed reduced high frequency acceleration in FM patients we cannot determine if this is due to enhanced coactivation. Altered muscle synergies are shown in FM patients [49], but future studies need to examine if they have a higher rate of coactivation in stability tasks and whether this can help to explain undue muscle fatigue commonly reported by FM patients.

There were no significant differences in normalized power in the middle frequency band (4–7 Hz) between FM patients and HCs. This component is proposed to be limb specific [50], of mechanical and reflexive origin, and is sensitive to load [12,13,17,18]. We found that arm girth, as an indication of limb mass, was positively associated with low frequency oscillations. Although the greater arm girth among patients may explain the FM patients’ shift towards lower frequencies in the unloaded condition, the additional loading did not lead to a consistent shift towards lower frequencies. Thus, the difference in arm volume does not explain the group difference in the power spectrum distribution.

### Muscle strength

The FM patients were significantly weaker than HCs in the shoulder abductors, but not in the elbow flexors. Our results add to the inconclusive literature concerning upper body muscle strength in FM [7,51-53]. FM patients generally have more pain in shoulder and neck muscles compared with arm muscles [54]. Pain or fear of pain may therefore explain the present findings of reduced MVC in shoulder abduction

The observed difference in power distribution between FM patients and HCs in the limb stability tasks were independent of load, indicating that the results are not due to reduced muscle strength. The use of absolute load instead of load relative to MVC will therefore be of little importance to our results. It has earlier been shown that reduced force steadiness in patients with chronic neck pain was not due to reduced strength [28].

## Conclusions

We have shown that limb stability during both loaded and unloaded elbow flexion and shoulder abduction position tasks is not significantly altered in FM patients. However, upper limb oscillations in FM patients showed a strong preponderance of normalized low frequency power compared to HCs. This difference does not seem to arise from instability or lower muscle strength in FM patients. We conclude that FM patients have a different motor control strategy to maintain limb stability in upper limb position tasks compared to HCs.

## Competing interest

The authors declare that they have no competing interest.

## Authors’ contribution

EMB participated in the design of the study, the collection of data, analysis and interpretation of data, and drafting the manuscript. KR participated in the design of the study, the interpretation of data, and critical revising of the manuscript. TOJ participated in the medical examination of the patients and critical revising of the manuscript. PJM participated in the design of the study, the interpretation of data, and drafting and critical revising of the manuscript. All authors read and approved the final manuscript.

## Pre-publication history

The pre-publication history for this paper can be accessed here:

http://www.biomedcentral.com/1471-2474/13/186/prepub
